# Supplementary material for: Pathway-Centric Comparative Molecular Profiling of Sézary Syndrome and Primary Cutaneous CD8+ Aggressive Epidermotropic Cytotoxic T-Cell Lymphoma via Conversational Artificial Intelligence
Source: Cancers (Basel). 2026 Apr 27;18(9):1387. doi: 10.3390/cancers18091387 (PMC13162890; doi:10.3390/cancers18091387)
Supplement: Supplementary file 1 [file cancers-18-01387-s001.zip › cancers-4292665-supplementary.pdf]

Supplementary Tables and Figures:

Table S1. Gene-level comparison of borderline-significant somatic mutation frequencies between Sézary syndrome and PCAECTCL cohorts.

Gene	Sezary Syndrome Mutated n (%)	Sezary Syndrome Wild-Type n (%)	PCAECTCL Mutated n (%)	PCAECTCL Wild-Type n (%)	p-value
ERBB2	0 (0%)	26 (100%)	3 (23%)	10 (77%)	**0.03129**

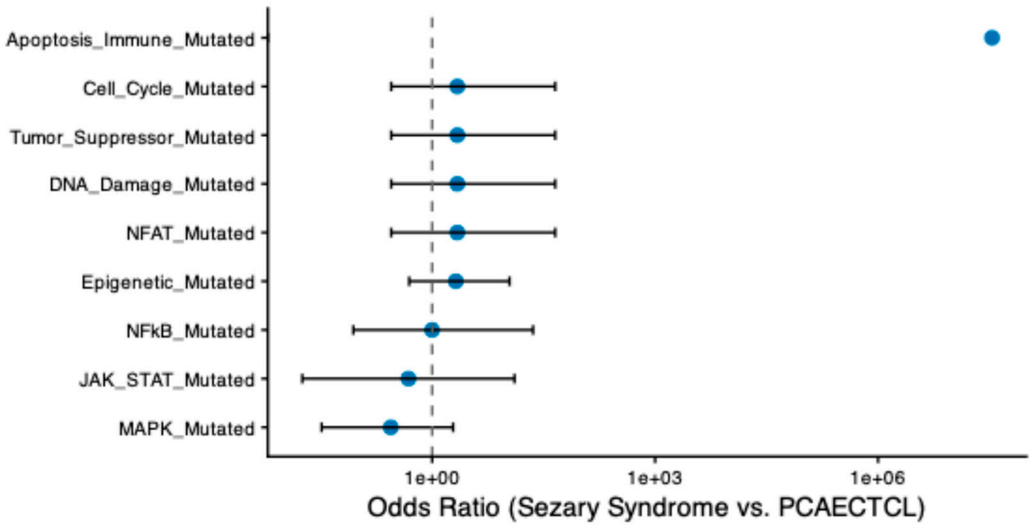

Figure S1. Comparative odds ratios of pathway-level alterations between Sézary syndrome and PCAECTCL.

This forest plot displays the odds ratios and associated confidence intervals for pathway-level mutation frequencies comparing Sézary syndrome (SS) to primary cutaneous CD8$^+$ aggressive epidermotropic cytotoxic T-cell lymphoma (PCAECTCL). Values greater than one indicate relative enrichment of pathway alterations in SS, whereas values less than one reflect higher frequencies in PCAECTCL. Pathways related to epigenetic regulation, tumor suppressor function, cell-cycle control, NFAT signaling, and DNA damage response demonstrate a consistent shift toward higher representation in SS, suggesting a greater contribution of transcriptional and regulatory disruption in this subtype. In contrast, MAPK and JAK/STAT signaling pathways trend below unity, indicating relatively increased involvement in PCAECTCL. NF-κB pathway alterations cluster near the null value, consistent with comparable frequencies across both cohorts. Notably, apoptosis and immune regulatory pathways show the largest deviation toward SS, driven by their presence in SS tumors and absence in PCAECTCL. The distribution of odds ratios highlights distinct pathway-level biases between these aggressive CTCL subtypes, reinforcing differences in their underlying molecular programs.

PCLO : [Somatic Mutation Rate: 19.23%]
NM_033026

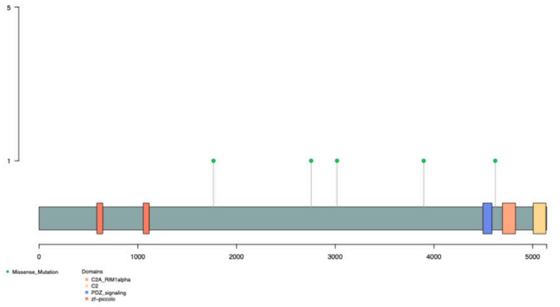

AHNAK2 : [Somatic Mutation Rate: 15.38%]
NM_138420

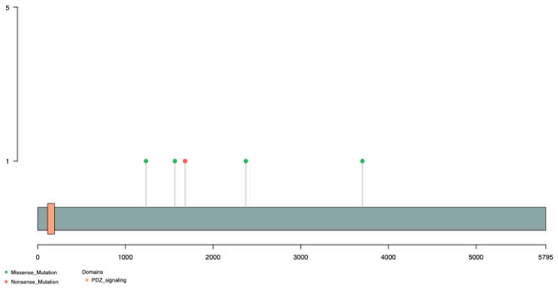

FBN2 : [Somatic Mutation Rate: 15.38%]
NM_001999

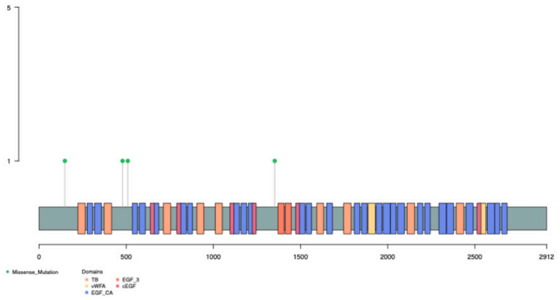

TP53 : [Somatic Mutation Rate: 15.38%]
NM_000546

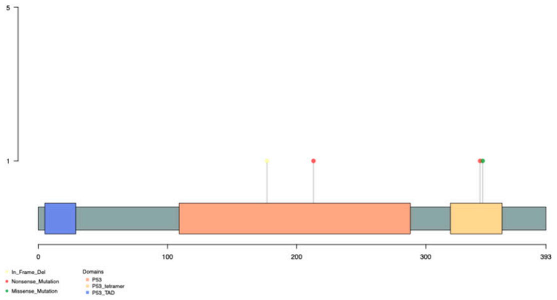

DNAH5 : [Somatic Mutation Rate: 11.54%]
NM_001369

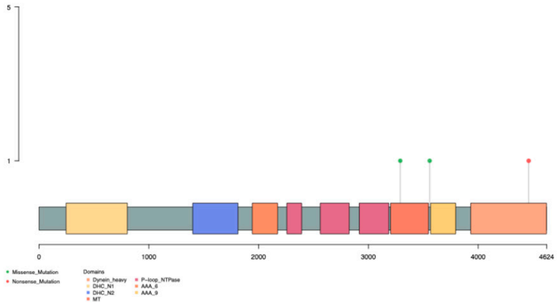

FAM47C : [Somatic Mutation Rate: 11.54%]
NM_001013736

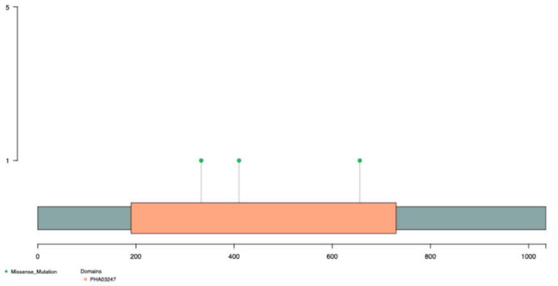

LRP1B : [Somatic Mutation Rate: 11.54%]
NM_018557

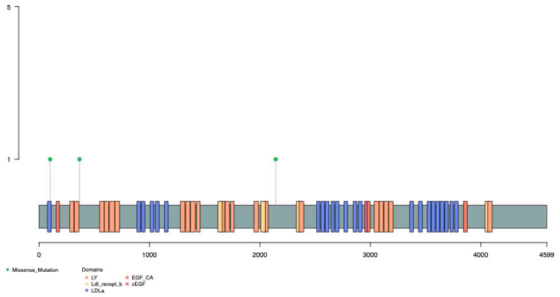

KIT : [Somatic Mutation Rate: 11.54%]
NM_000222

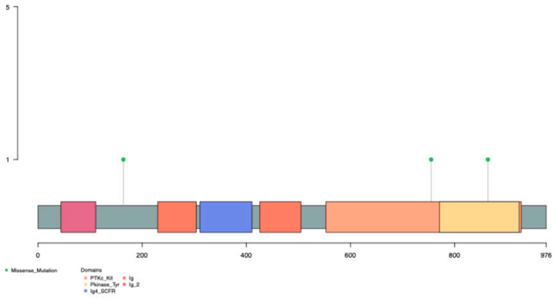

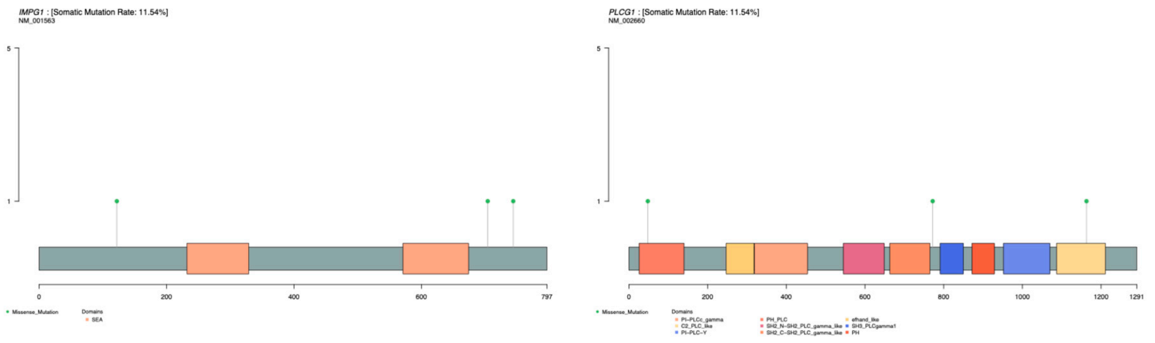

Figure S2. Lollipop plots illustrating the distribution of somatic mutations in the top 10 recurrently mutated genes in the Sézary syndrome cohort. This figure presents lollipop plots depicting the positional distribution of somatic mutations across the protein sequences of the ten most frequently mutated genes identified in the Sézary syndrome (SS) cohort. Each panel corresponds to a single gene, and each lollipop marker represents an individual mutation event. The horizontal axis denotes the amino acid position along the protein sequence, while the vertical lollipop height reflects the number of tumor samples harboring mutations at that site. Colored markers indicate the type of somatic alteration, and annotated protein domains are shown along the protein structure to contextualize mutation locations. Together, these plots highlight mutation clustering and potential hotspot regions across the most frequently altered genes in SS, providing insight into candidate functional domains that may contribute to disease pathogenesis.

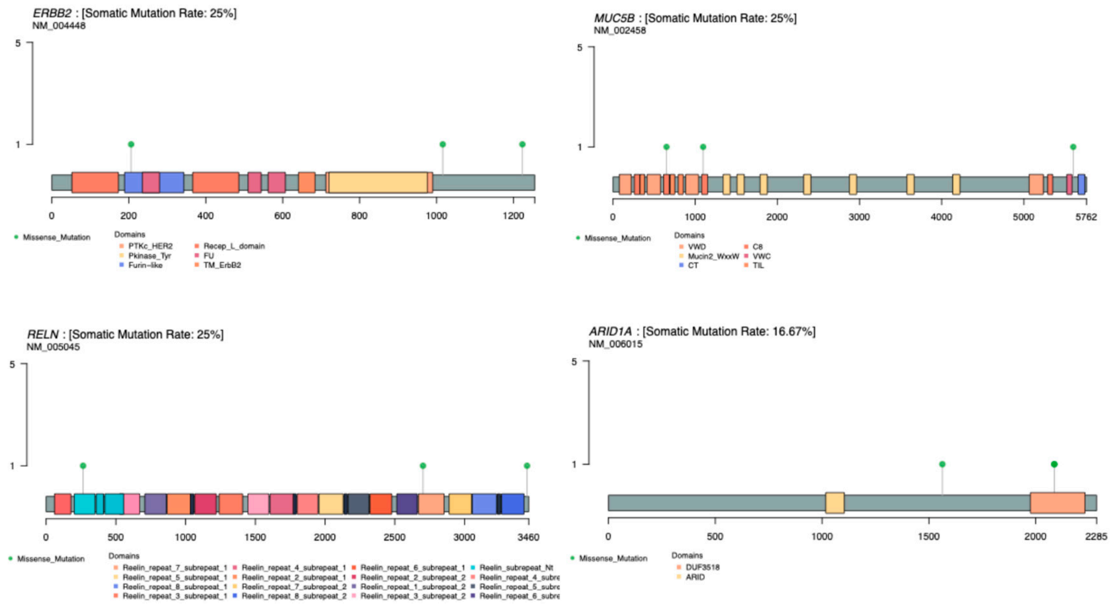

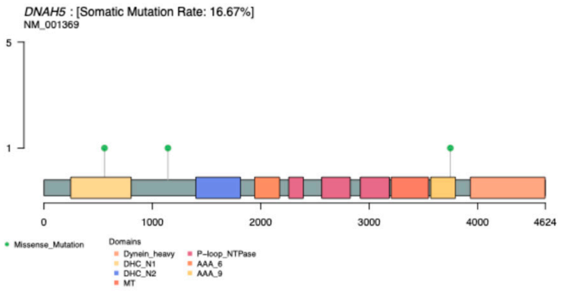
DNAH5 : [Somatic Mutation Rate: 16.67%]
NM_001369
5
1
0
1000
2000
3000
4000
4624
Missense_Mutation
Domains
Dynein_heavy
DHC_N1
DHC_N2
MT
P-loop_NTPase
AAA_6
AAA_9

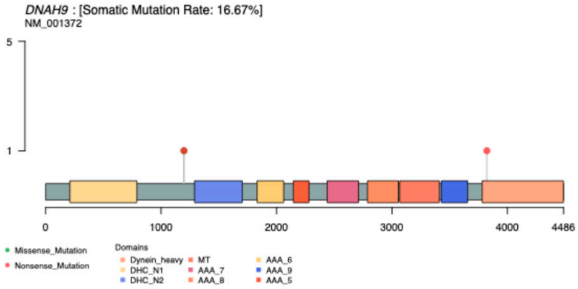
DNAH9 : [Somatic Mutation Rate: 16.67%]
NM_001372
5
1
0
1000
2000
3000
4000
4486
Missense_Mutation
Nonsense_Mutation
Domains
Dynein_heavy
DHC_N1
DHC_N2
MT
AAA_7
AAA_8
AAA_6
AAA_9
AAA_5

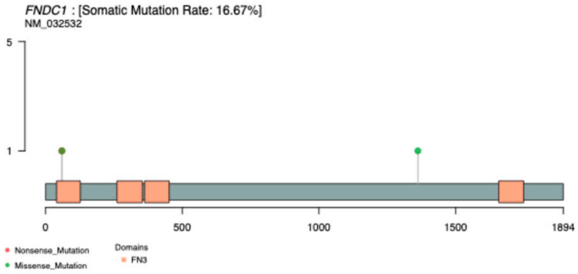
FNDC1 : [Somatic Mutation Rate: 16.67%]
NM_032532
5
1
0
500
1000
1500
1894
Nonsense_Mutation
Missense_Mutation
Domains
FN3

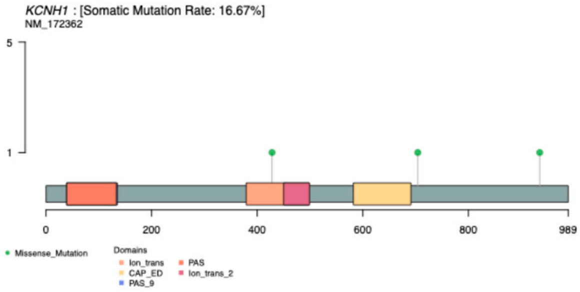
KCNH1 : [Somatic Mutation Rate: 16.67%]
NM_172362
5
1
0
200
400
600
800
989
Missense_Mutation
Domains
Ion_trans
CAP_ED
PAS_9
PAS
Ion_trans_2

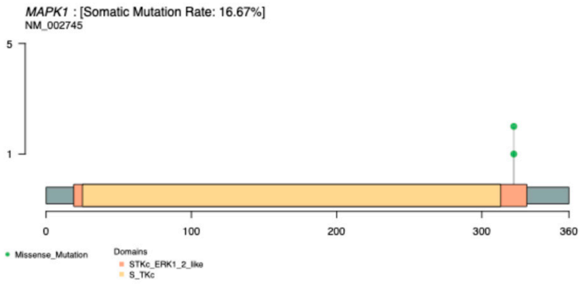
MAPK1 : [Somatic Mutation Rate: 16.67%]
NM_002745
5
1
0
100
200
300
360
Missense_Mutation
Domains
STKc_ERK1_2_like
S_TKc

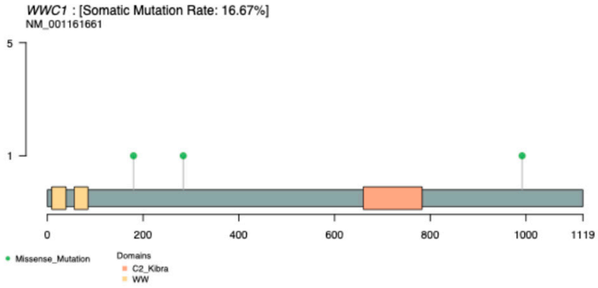
WWC1 : [Somatic Mutation Rate: 16.67%]
NM_001161661
5
1
0
200
400
600
800
1000
1119
Missense_Mutation
Domains
C2_Kibra
WW

Figure S3. Lollipop plots illustrating the distribution of somatic mutations in the top 10 recurrently mutated genes in the Primary Cutaneous CD8⁺ Aggressive Epidermotropic Cytotoxic T-Cell Lymphoma (PCAECTC) cohort. This figure presents lollipop plots depicting the positional distribution of somatic mutations across the protein sequences of the ten most frequently mutated genes identified in the PCAECTC. Each panel corresponds to an individual gene, with lollipop markers representing mutation events observed across tumor samples. The horizontal axis indicates the amino acid position along the protein sequence, while the vertical height of each lollipop reflects the number of samples harboring mutations at that specific site. Colored markers denote different mutation types, and annotated protein domains provide structural context for the observed alterations. These visualizations highlight mutation distribution patterns and potential hotspot regions across recurrently altered genes in PCAECTC, offering insight into candidate functional regions that may contribute to disease biology.

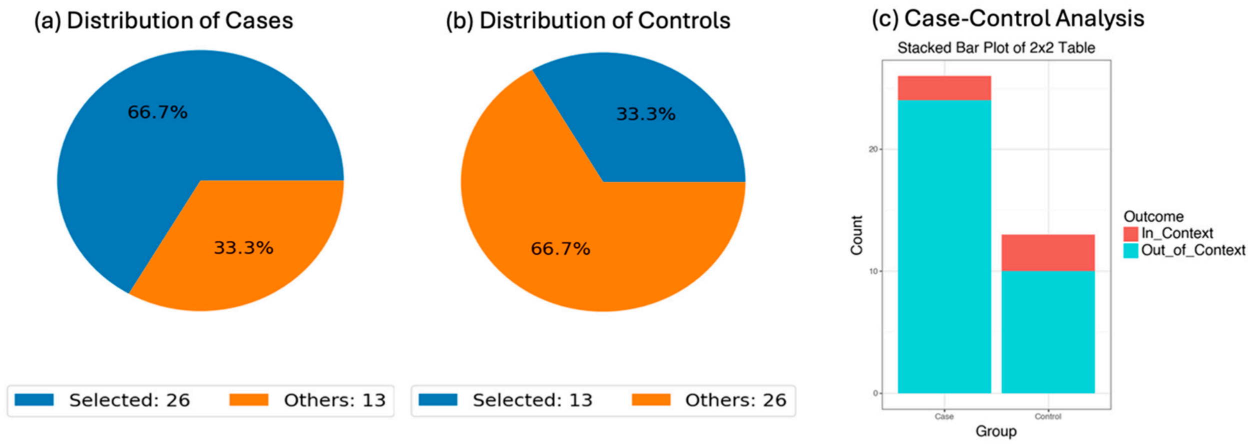

Figure S4. Conversational AI-guided case–control evaluation of MAPK pathway alterations in Sézary syndrome and PCAECTCL. This figure presents a conversational AI-assisted analytical workflow designed to assess differences in the prevalence of MAPK pathway alterations between Sézary syndrome (SS) and primary cutaneous CD8⁺ aggressive epidermotropic cytotoxic T-cell lymphoma (PCAECTCL). The case cohort consisted of SS samples (n = 26), while the control cohort included PCAECTCL samples (n = 13), defined through structured query logic within the AI-driven platform. Panels (a) and (b) display the proportional representation of selected samples relative to the full dataset for each cohort, illustrating the relative cohort sizes and composition. Panel (c) summarizes the distribution of MAPK-mutated ("in-context") and non-mutated ("out-of-context") samples using a stacked bar plot derived from a 2×2 contingency framework. MAPK alterations were identified in a subset of tumors in both groups, with a slightly higher proportion observed in PCAECTCL compared to SS. Statistical evaluation using Fisher's exact test yielded a non-significant association ($p = 0.397$), with an odds ratio of 0.278 and a wide confidence interval, reflecting limited statistical power. Overall, this analysis demonstrates how conversational AI frameworks can be leveraged to rapidly construct and visualize subtype-specific comparisons of pathway alterations, supporting exploratory interrogation of genomic differences across CTCL subtypes.

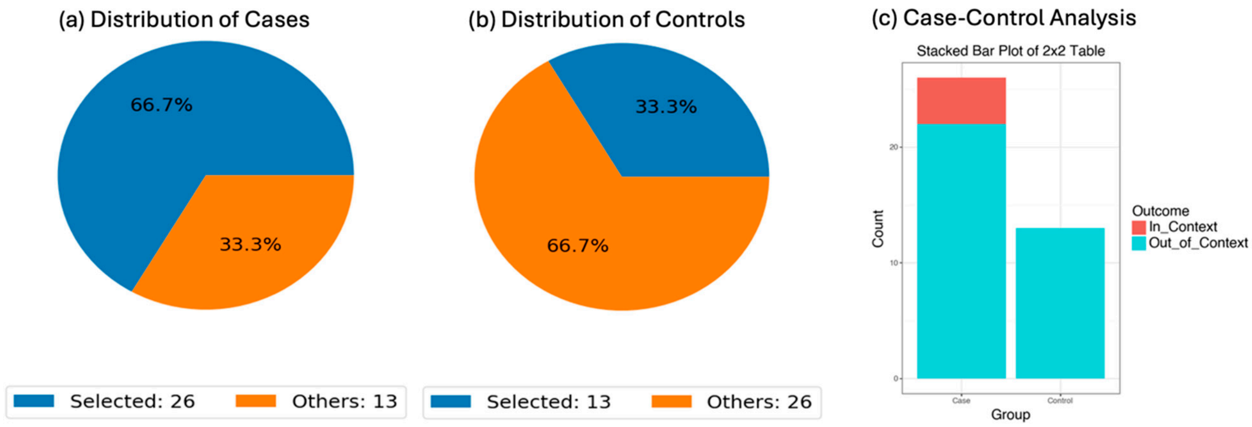

Figure S5. Conversational AI-enabled case–control assessment of pathway alteration prevalence between Sézary syndrome and PCAECTCL. This figure illustrates a conversational AI-driven case–control analysis evaluating differences in the prevalence of selected molecular alterations between Sézary syndrome (SS) and primary cutaneous CD8^{+} aggressive epidermotropic cytotoxic T-cell lymphoma (PCAECTCL). Cohorts were defined within the AI framework using structured query criteria, with SS samples designated as the case group (n = 26) and PCAECTCL samples as the control group (n = 13). Panels (a) and (b) depict the proportional representation of selected samples relative to the full dataset for each cohort, highlighting the relative sizes and composition of the groups included in the analysis. Panel (c) presents a stacked bar plot summarizing the number of samples with ("in-context") and without ("out-of-context") the specified molecular alteration using a 2×2 contingency structure. The distribution of altered versus non-altered samples appears comparable between cohorts, and statistical testing did not reveal a significant difference in prevalence, consistent with overlapping mutation frequencies. This visualization demonstrates how conversational AI frameworks can rapidly construct and interpret case–control comparisons, enabling efficient exploration of subtype-specific genomic patterns in CTCL.

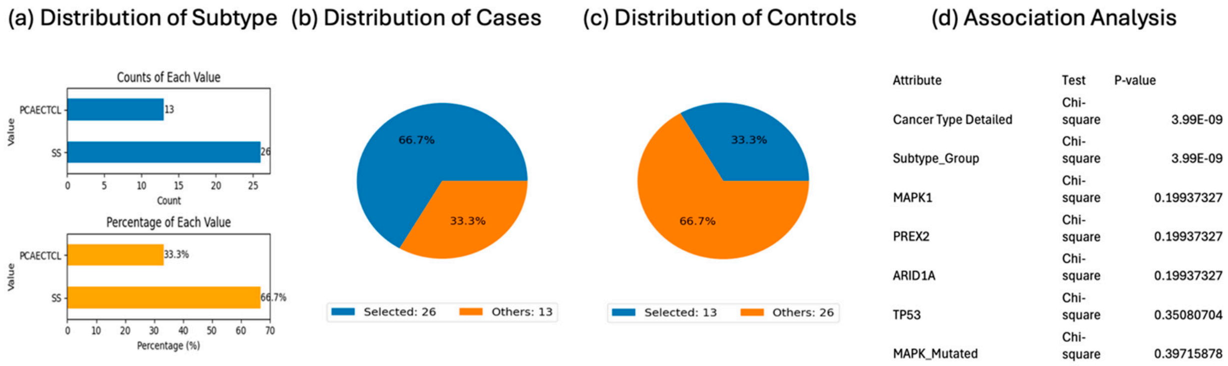

Figure S6. Conversational AI-assisted analysis of clinical and molecular feature associations between Sézary syndrome and PCAECTCL.

This figure summarizes a conversational AI-driven case–control framework used to explore associations between clinical attributes and selected genomic features in Sézary syndrome (SS) and primary cutaneous CD8^{+} aggressive epidermotropic cytotoxic T-cell lymphoma (PCAECTCL). The case cohort consisted of SS samples (n = 26), while the control cohort included PCAECTCL samples (n = 13), defined using structured query logic within the AI platform. Panel (a) displays the distribution of subtype classification across the dataset, illustrating both absolute counts and relative proportions for each group. Panels (b and c) present pie charts depicting the proportion of selected versus non-selected samples within the case and control cohorts, reflecting their contribution to the overall dataset. Panel (d) summarizes statistical associations between clinical variables and molecular

features using chi-square testing. As expected, subtype-defining variables such as “Cancer Type Detailed” and “Subtype_Group” show highly significant differences between cohorts ($P \approx 3.99\times10^{-9}$). In contrast, individual gene-level features, including MAPK1, PREX2, ARID1A, TP53, and aggregated MAPK pathway status, do not demonstrate statistically significant differences, consistent with the broader observation of comparable mutation burdens but divergent pathway organization. Overall, this figure highlights how conversational AI can integrate clinical and genomic variables to systematically evaluate subtype-associated patterns in CTCL.
